# A Differential Effect of Indian Ocean Dipole and El Niño on Cholera Dynamics in Bangladesh

**DOI:** 10.1371/journal.pone.0060001

**Published:** 2013-03-29

**Authors:** Masahiro Hashizume, Luis Fernando Chaves, A. S. G. Faruque, Md Yunus, Kim Streatfield, Kazuhiko Moji

**Affiliations:** 1 Institute of Tropical Medicine (NEKKEN) and the Global Center of Excellence program, Nagasaki University, Nagasaki, Japan; 2 Programa de Investigación en Enfermedades Tropicales, Escuela de Medicina Veterinaria, Universidad Nacional, Heredia, Costa Rica; 3 International Centre for Diarrhoeal Disease Research, Bangladesh, Dhaka, Bangladesh; 4 Research Institute for Humanity and Nature, Kyoto, Japan; Beijing Institute of Microbiology and Epidemiology, China

## Abstract

**Background:**

A stationary (i.e., constant through time) association between El Niño Southern Oscillation (ENSO), the Indian Ocean Dipole (IOD) and epidemics of cholera in Bangladesh has been widely assumed. However, whether or not elements of the local climate that are relevant for cholera transmission have stationary signatures of the IOD on their dynamics over different time scales is still not clear. Here we report results on the time-varying relationships between the various remote and local environmental drivers and cholera incidence in Bangladesh.

**Methodology/Principal Findings:**

We performed a cross wavelet coherency analysis to examine patterns of association between monthly cholera cases in the hospitals in Dhaka and Matlab (1983–2008) and indices for both IOD and ENSO. Our results showed that the strength of both the IOD and ENSO associations with cholera hospitalizations changed across time scales during the study period. In Dhaka, 4-year long coherent cycles were observed between cholera and the index of IOD in 1988–1997. In Matlab, the effect of ENSO was more dominant while there was no evidence for an IOD effect on cholera hospitalizations.

**Conclusions/Significance:**

Our results call for the consideration of non-stationary, possibly non-linear, patterns of association between cholera hospitalizations and climatic factors in cholera epidemic early warning systems.

## Introduction

Cholera remains a major public health problem in many places, including Bangladesh, India, and a number of countries in Africa and South America [1]. *Vibrio cholerae*, the bacterium that causes the disease, is known to inhabit riverine, estuarine and coastal ecosystems with specific abiotic (e.g., temperature, sunlight, pH, salinity) and biotic components (e.g., phytoplankton, aquatic plants, and copepod zooplankton) [2]–[4]. *V. cholerae* has an increased growth rate in aquatic environments with warmer temperatures, particularly when combined with a high pH and blooms of phytoplankton, aquatic plants or algae [5]–[7].

It has been reported that the El Niño-Southern Oscillation (ENSO) plays a role in the interannual variation of endemic cholera in Bangladesh [6], [8]–[10]. Sea surface temperature (SST) and sea surface height (SSH) in the Bay of Bengal have been proposed to influence the incidence of cholera in Dhaka [6], [11]–[13]. The strong correlation between SST in the Bay of Bengal and outbreaks of cholera may occur because the warm waters along the coast, coupled with phytoplankton blooms driven by warm ocean temperatures, are favorable for *V. cholerae* multiplication [12], [14]. SSTs over the entire basin of the Indian Ocean are uniformly modulated by the ENSO after a few months lag [15].

The Indian Ocean Dipole (IOD) is another climate mode that arises from ocean-atmosphere interactions that cause interannual climate variability in the tropical Indian Ocean [16], [17]. A positive IOD indicates SST anomalies, with warmer than normal SSTs over the western basin and cooler than usual SSTs in the eastern basin near Sumatra. Conversely, a negative IOD indicates warmer than normal SSTs over the eastern basin and cooler than usual SSTs in the western tropical Indian Ocean. Although the extent to which the IOD is independent of ENSO has been debated [18], there is growing evidence that this air-sea interaction is specific to the Indian Ocean [19]–[21]. The IOD has been reported to affect regional ocean climate [22]. IOD events strongly influence sea level variations in the Bay of Bengal and sea level anomalies in the northern Bay may influence flooding and outbreaks of cholera in Bangladesh [23]. The IOD also plays an important role as a modulator of the Indian monsoon rainfall [24]–[27]. Rainfall and associated river levels have also been reported to influence cholera patterns in Bangladesh [28], [29] and a short-term temporal association between IOD and cholera incidence in Bangladesh has been reported [30]. These studies assumed that the association between cholera incidence and ENSO and IOD was consistent over the study period. However, whether elements of the local climate that are relevant for cholera transmission have stationary (i.e., constant through time) associations with the ENSO and IOD over time is still not clear, and previous findings based on this assumption can be improved by assuming a non-stationary (not consistent overtime) association. This is of special interest because the association between the IOD, ENSO and Indian summer monsoon rainfall has been reported to vary over time [27]. Cholera is still a major health risk in many areas of the world and a better understanding of its sensitivity to climate may contribute to the development of a reliable climate-based prediction system of cholera epidemics that could potentially lead to an improvement in the currently existing disease control programme in Bangladesh and other countries where cholera is endemic. The objective of the present study is to explore the time-varying relationships between global (i.e., IOD, ENSO) and local (i.e., temperature, rainfall, river level and SST in the Bay of Bengal) environmental drivers and cholera hospitalizations in Bangladesh, using cross wavelet analysis.

Wavelet analysis is useful for epidemiological time series analyses mainly because of the non-stationarity of associations between the disease dynamics and exposure covariates [31]. Wavelet analysis is a method that has been used to determine whether the presence of a particular periodic cycle at a given time in a disease incidence corresponds to the presence of the same periodical cycle at the same time in an exposure covariate [31]. Wavelet analysis has increasingly been used in epidemiology to explore the spatial and temporal dynamics of diseases [32]–[36].

## Methods

### Data

#### Hospital surveillance

**Figure 1:**
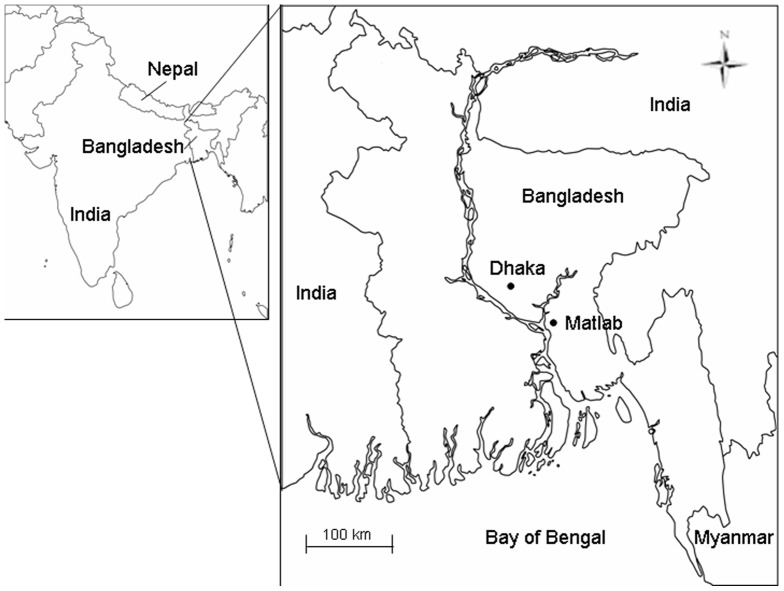
Map of the study sites. The left panel shows the location of Bangladesh in South Asia (the Indian subcontinent) and the right panel highlights the location of Dhaka and Matlab, the study sites in Bangladesh.

The primary outcome for this study was the monthly number of patients with cholera who attended the International Centre for Diarrhoeal Disease Research, Bangladesh (ICDDR,B) hospitals in Dhaka and Matlab (Figure 1). ***Dhaka***: The ICDDR,B hospital serves a large urban population within the city of Dhaka and provides free treatment for more than 100,000 cases of diarrhea each year. A surveillance system was established at the ICDDR,B in 1979 to systematically sample children and adults with diarrheal illnesses [37]. Up to 1995, every 25^th^ patient was enrolled in the surveillance system, and since 1996 every 50^th^ patient has been enrolled. **Matlab**. Matlab is a rural and riverine delta area situated approximately 57 km southeast of Dhaka. Every hospital visit of patients residing within the area covered by the Health and Demographic Surveillance System (HDSS), which consists of 142 villages with populations over 220,000, was registered in the hospital surveillance system [38].

For each diarrhea patient of the ICDDR,B hospitals in Dhaka and Matlab, the stool was microbiologically examined to identify common enteric pathogens including *V. cholerae*. The details of the laboratory procedures were described in a previous study [37]. A patient was enrolled in the study when *V. cholerae* of the serogroups O1 or O139 was identified in the stool specimen, regardless of the presence of other pathogens. From the surveillance data that had been collected over a 26-year period (January 1983 to December 2008) for Dhaka and 28-year period (January 1981 to December 2008) for Matlab, we retrieved the date of the hospital visit and the pathogens identified in each stool specimen. From these data, the monthly cholera cases in Dhaka and Matlab were counted separately and used for analysis.

#### Ocean climate data

The strength of the IOD was measured by the dipole mode index (DMI), defined as the difference in SST between the western (10°S–10°N, 50–70°E) and eastern (10°S–0°, 90–110°E) tropical Indian Ocean [16]. The monthly average DMI values were obtained from the Japan Agency for Marine-Earth Science and Technology (www.jamstec.go.jp/frcgc/research/d1/iod/). The DMI values were calculated using the SST data from the HadISST dataset (http://www.metoffice.gov.uk/hadobs/hadisst/). The base period for calculating anomalies was 1958–2008. These values were standardized to zero mean and unit standard deviation. The strength of the ENSO was measured by monthly average SSTs in the Niño 3 region (5°S–5°N, 150–90°W) (Nino3) in the Pacific Ocean that we derived from the National Oceanic and Atmospheric Administration (NOAA) Climate Prediction Center data (http://www.cpc.ncep.noaa.gov). We examined the association between DMI and monthly average SST in the northern Bay of Bengal to gain some insight into the causal pathways linking the IOD to the number of cholera cases. Mean monthly SSTs in the Bay of Bengal (20–21°N, 90–91°E) were derived from the NOAA Optimum Interpolation Sea Surface Temperature dataset [39], [40].

### Meteorological and hydrological data

The daily rainfall and maximum and minimum temperature data for Dhaka and Matlab were obtained from the Bangladesh Meteorological Department. The data for the daily river levels of the Brigonga (Millbarrak in Dhaka) and Danagoda (Matlab Bazaar) rivers were obtained from the records maintained by the Bangladesh Water Development Board. The monthly means for the maximum temperature (°C) and maximum river level (m), and the total monthly rainfall (mm) were calculated from the daily records. Missing values (17 [5.4%] in Dhaka and 6 [1.9%] in Matlab) for river levels were imputed using the average values of the same month in the other years over the whole time series.

### Statistical analysis

#### Temporal patterns of association in the time-frequency domain

Temporal patterns of association in the time series were studied using the continuous wavelet transform [33] [31]. Specifically, cross wavelet coherency analysis was used to determine whether the presence of a particular frequency at a given time in cholera corresponded to the presence of the same frequency at the same time in a climate covariate. A coherence analysis is similar to a correlation analysis in the sense that it is normalized between 0 and 1, where 0 corresponds to the total absence of cycles with the same period in the analyzed time series, and 1 corresponds to the presence of cycles with exactly the same periods in the analyzed time series [41]. Briefly, for the two time series whose association is under study, the cross wavelet spectrum (CWS) is estimated through time for a series of frequencies (or period, i.e., 1/frequency). The CWS is then normalized by the product of each time series square root transformed wavelet power spectra. Thus, to make an analogy between coherency and correlation, the CWS can be seen as equivalent to covariance which is normalized by the power spectra of two variables in the same way that covariance is normalized by the product of the standard deviation of two variables when their correlation is estimated. When the magnitude of the CWS is similar to the normalized product of the time series spectra the ratio is equal to 1, indicating that the two studied time series have cycles with the same period.

We also estimated the cone of influence, where inferences from the wavelet analysis outside the cone of influence are not valid because of the manipulations that were performed to generate the wavelet spectrum in the absence of data for larger period frequencies [31]. Cross wavelet coherence significance was estimated using the method described by Maraun and Kurths [42] for a minimum time scale, S_0_ = 6 months (i.e., the minimum period of interest in the cycles studied with the cross wavelet analysis was 6 months), a total smoothing window of 31 (i.e., W = 15) and parameter W_0_ = 6 (dimensionless parameter of the Morlet Wavelet). Further details about cross wavelets and software are described in Maraun and Kurths [42] and Chaves and Pascual [33]. To analyze the association between cholera hospitalizations and environmental factors, we classified the data into two groups, one for the *V. cholerae* El Tor O1 biotype strain and one for the O139 Bengal strain.

We did not stratify the analysis by age, because the seasonal patterns were the same and there was no likely reason to expect different cholera-climate associations between the age groups at interannual time scales.

The associations between DMI, Nino3 and monthly average temperature, rainfall and SST in the northern Bay of Bengal were also examined by wavelet coherence analysis. All the analyses were performed with R software (version 2.0).

## Results

**Figure 2:**
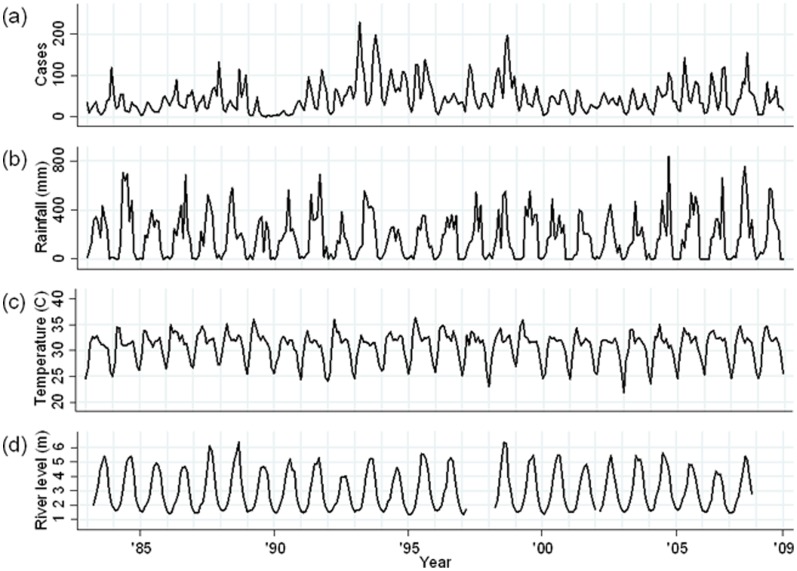
Monthly time series data for Dhaka (January 1983–December 2008). (a) Total cholera cases; (b) Monthly rainfall (mm); (c) Monthly average of daily maximum temperature (°C); (d) Monthly average of daily mean river level (m) of Brigonga river (the data for 2008 for Millbarrak in Dhaka was missing).

**Figure 3:**
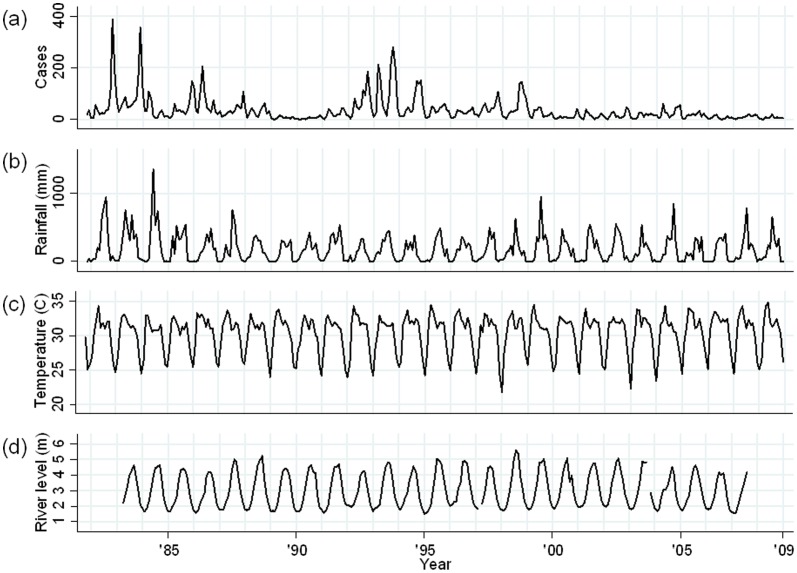
Monthly time series data for Matlab (November 1981–December 2008). (a) Total cholera cases; (b) Monthly rainfall (mm); (c) Monthly average of daily maximum temperature (°C); (d) Monthly average of daily mean river level (m) of Danagoda river (the data for 2008 and before January 1983 for Matlab Bazar was missing).

The time series for the number of cholera patients per month, by serogroup and biotype, ambient temperature, rainfall and river level during the study period are shown for Dhaka (Figure 2) and Matlab (Figure 3).

**Figure 4:**
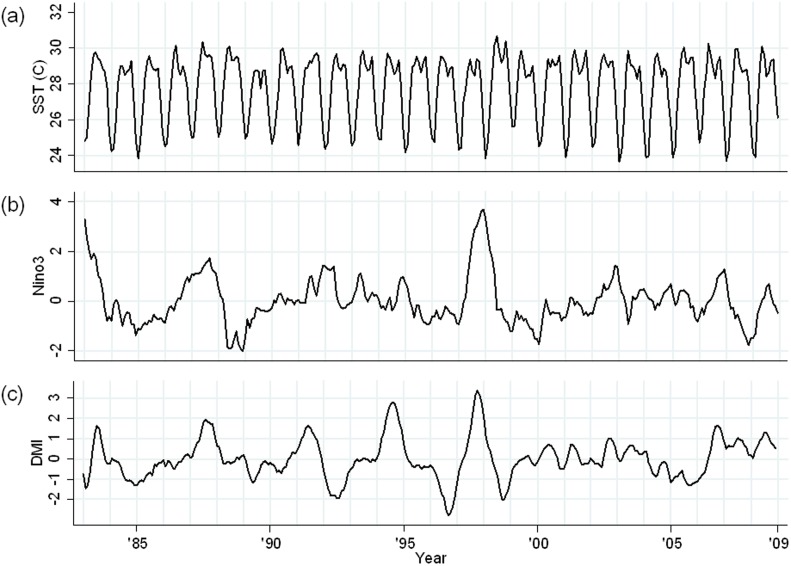
Monthly time series data for global climatic indices (November 1981–December 2008). (a) Sea surface temperature (SST) in the Bay of Bengal (°C); (b) Nino3; (c) Dipole mode index (DMI).

The time series for Dipole Mode Index (DMI), Nino3 and sea surface temperature (SST) during the same period are shown in Figure 4. Strong positive IOD events (indicated by large DMI values) occurred in 1994 and 1997 and, in these years, the DMI peaked in August and October, respectively. Strong ENSO events (indicated by large Nino3 index values) were observed in 1982–1983 and again in 1997–1998. In 1998, exceptionally high SSTs were observed and this preceded a sharp increase in the number of cholera hospitalizations in Dhaka.

**Figure 5:**
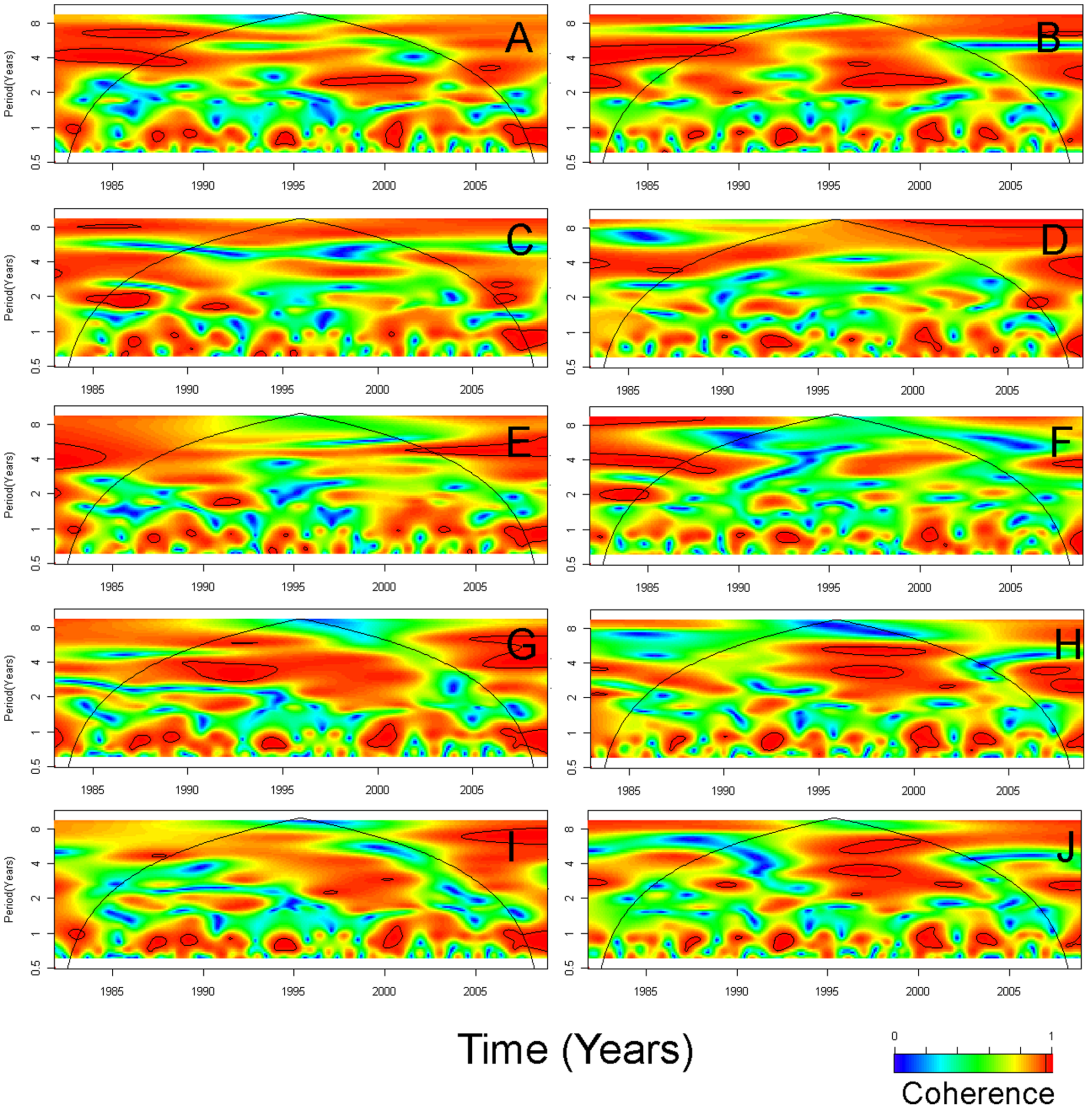
Cross wavelet coherence of global with local climatic time series. (A) Dipole mode index (DMI) and sea surface temperature (SST) in the Bay of Bengal (°C),(B) Nino3 and SST in the Bay of Bengal (°C); (C) Dhaka rainfall (mm) and DMI; (D) Dhaka rainfall (mm) and Nino3; (E) Matlab rainfall (mm) and DMI; (F) Matlab rainfall (mm) and Nino3; (G) Dhaka temperature (°C) and DMI; (H) Dhaka temperature (°C) and Nino3; (I) Matlab temperature (°C) and DMI; (J) Matlab temperature (°C) and Nino3. Red regions in the upper part of the plots indicate frequencies and times for which the two series share variability. The cone of influence (within which results are not influenced by the edges of the data) and the significant coherent time-frequency regions (p<0.05) are indicated by solid lines.

Figure 5 shows cross wavelet coherence of the global climatic time series (DMI and Nino3) with the local climatic time series (SST, rainfall and temperature). Red regions in the upper part of the plots in Figure 5 indicate frequencies and times for which the two series share variability. Biannual cycles of SSTs were associated with DMI and Nino3 between 1997 and 2000. In Dhaka, 3-year temperature cycles were associated with DMI in 1990–1995 and, in Dhaka and Matlab, the temperature cycles were associated with Nino3 in 1995–2000.

**Figure 6:**
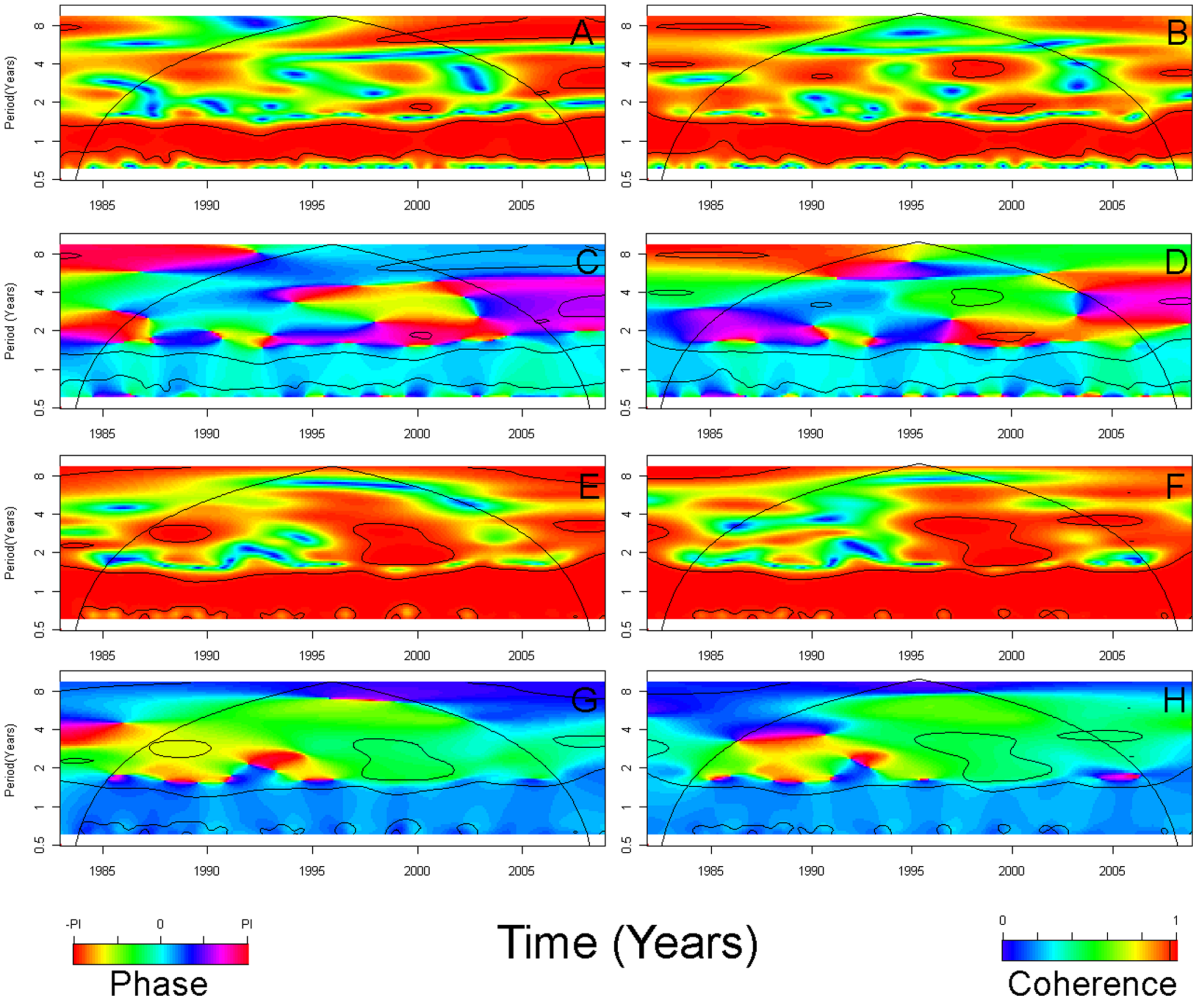
Cross wavelet analysis (CWA, coherence [C] and phase [P]) of sea surface temperature (SST) in the Bay of Bengal (°C) with local rainfall (mm) and temperature (°C). (A) CWAC of SST in the Bay of Bengal and Dhaka rainfall; (B) CWAC of SST in the Bay of Bengal and Matlab R; (C) CWAP of SST in the Bay of Bengal and Dhaka rainfall; (D) CWAP of SST in the Bay of Bengal and Matlab rainfall; (E) CWAC of SST in the Bay of Bengal and Dhaka temperature; (F) CWAC of SST in the Bay of Bengal and Matlab temperature; (G) CWAP of SST in the Bay of Bengal and Dhaka temperature; (H) CWAP of SST in the Bay of Bengal and Matlab temperature. In cross wavelet phase plots, colors correspond to different lags between the variability in the two series for a given time and frequency, measured in angles from -PI to PI. A value of PI corresponds to a lag of 16 months. Dhaka time series span from January 1983 to December 2008. Matlab time series span from November 1981 to December 2008.

Figure 6 shows the results of cross wavelet coherence and cross wavelet phase analysis of the SST in the Bay of Bengal with local rainfall and temperature. At the seasonal scale, rainfall and temperature in Dhaka and Matlab were strongly associated with SSTs and nearly synchronous as revealed by phase plots.

**Figure 7:**
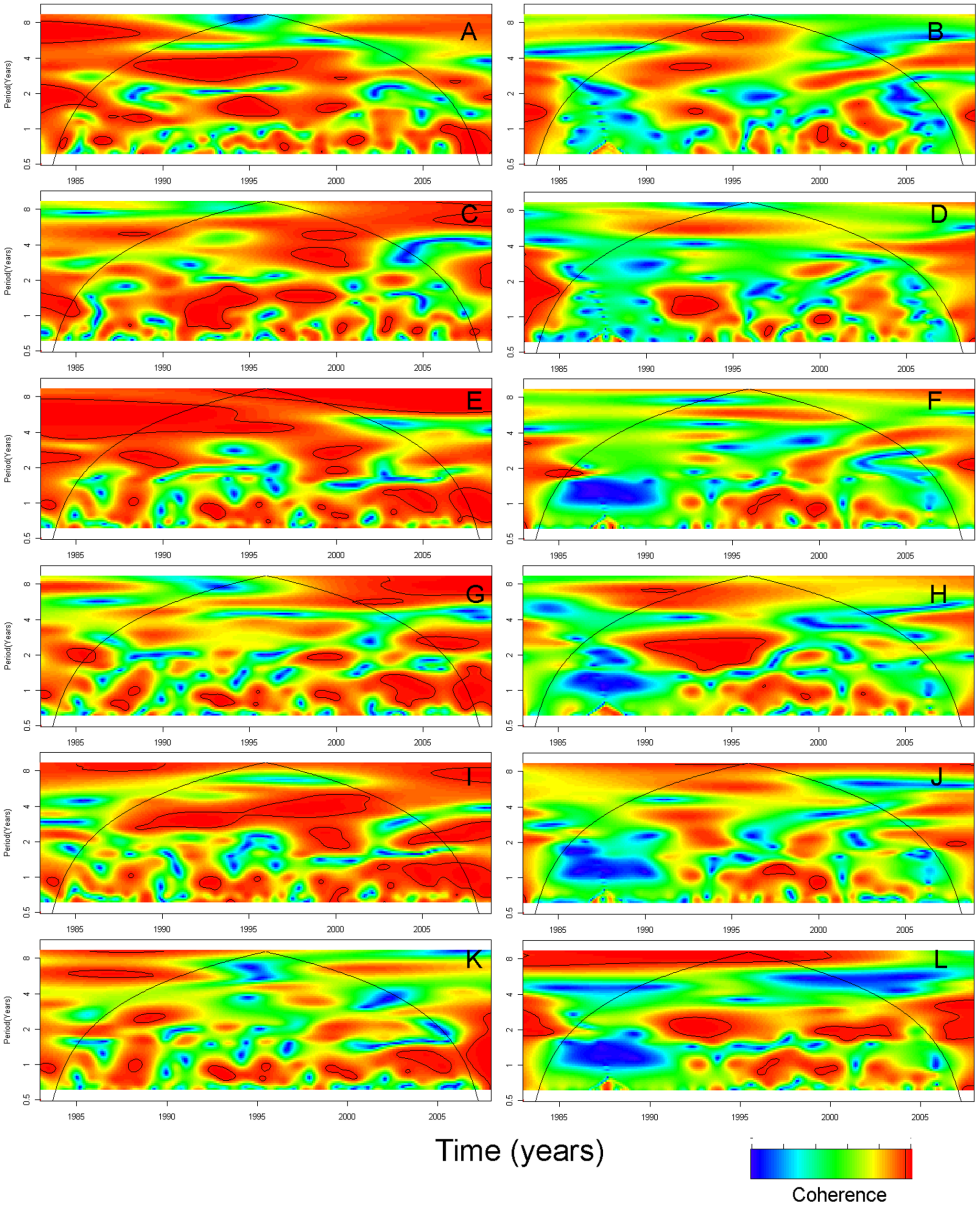
Cross wavelet coherence of global and local climatic time series with cholera in Dhaka by serotype O1 and O139. (A) Dipole mode index (DMI) and cholera O1; (B) DMI and cholera O139; (C) Nino3 and cholera O1; (D) Nino3 and cholera O139; (E) Sea surface temperature (SST) in the Bay of Bengal (°C) and cholera O1; (F) SST in the Bay of Bengal (°C) and cholera O139; (G) Rainfall (mm) and cholera O1; (H) Rainfall (mm) and cholera O139; (I) Temperature (°C) and cholera O1; (J) Temperature (°C) and cholera O139; (K) River level (m) and cholera O1; (L) River level (m) and cholera O139. Time series span from January 1983 to December 2008.

**Figure 8:**
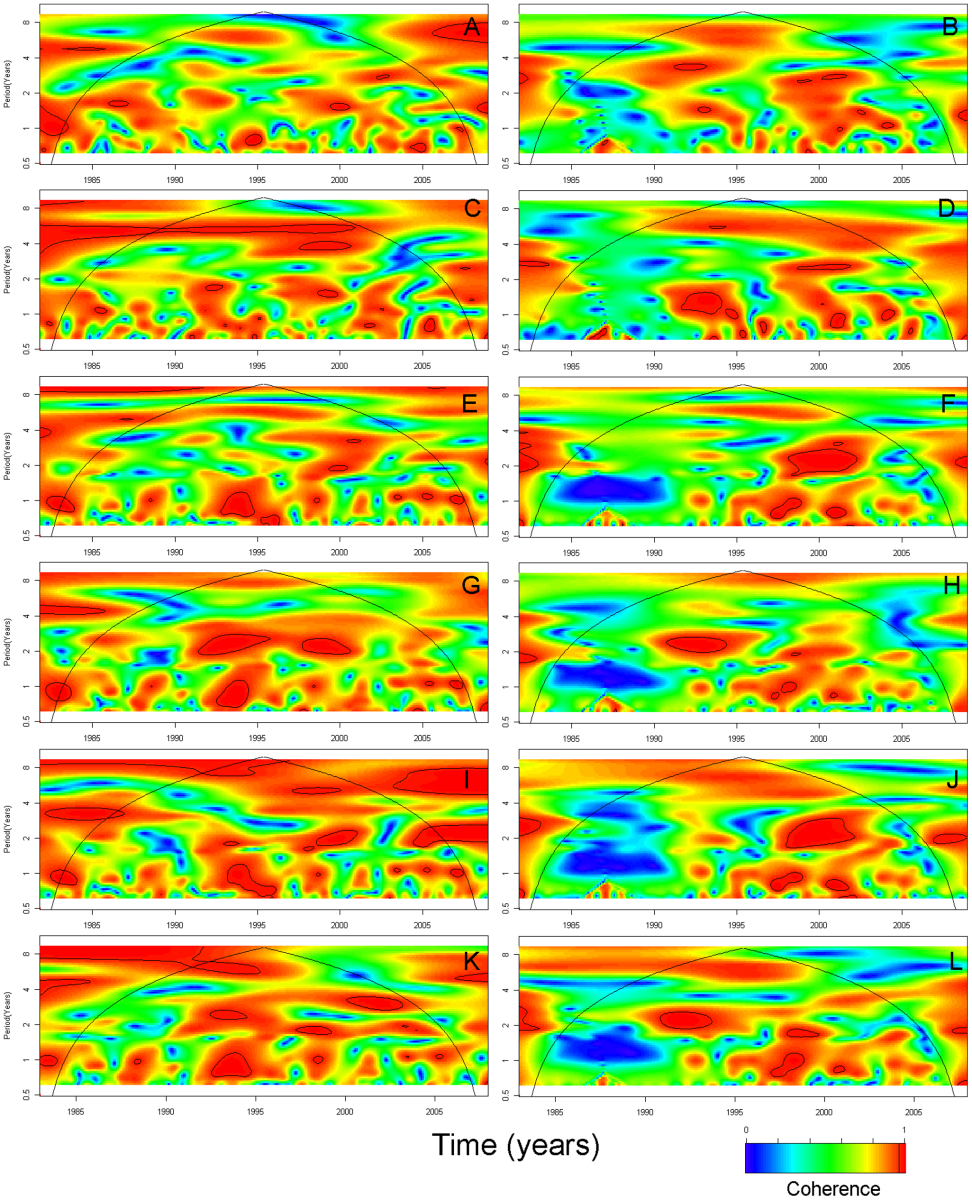
Cross wavelet coherence of global and local climatic time series with cholera in Matlab by serotype O1 and O139. (A) Dipole mode index (DMI) and cholera O1; (B) DMI and cholera O139; (C) Nino3 and cholera O1; (D) Nino3 and cholera O139; (E) Sea surface temperature (SST) in the Bay of Bengal (°C) and cholera O1; (F) SST in the Bay of Bengal (°C) and cholera O139; (G) Rainfall (mm) and cholera O1; (H) Rainfall (mm) and cholera O139; (I) Temperature (°C) and cholera O1; (J) Temperature (°C) and cholera O139; (K) River level (m) and cholera O1; (L) River level (m) and cholera O139. Time series span from November 1981 to December 2008, with the exception of river level that begins in January 1983.

Four-year long coherent cycles between O1 cholera and DMI were seen in 1988–1997 in Dhaka (Figure 7A) while the association with Nino3 was not consistent over time (3–4 year-cycles in 1998–2001 and 1–2 year-cycles in 1991–2000) (Figure 7C). Four- to 8-year long coherent cycles were observed between O1 cholera and SSTs in 1988–1997 (Figure 7E). Three- to five-year long coherent cycles were seen between O1 cholera and temperature in 1988–2002 (Figure 7I). Some non-stationary (non-consistent over time) seasonal cycles were seen between O1 cholera and rainfall and river level in Dhaka (Figure 7G and 7K). In Matlab, wavelet coherency between O1 cholera and DMI was less obvious (Figure 8A); however, 5–6-year long coherent cycles between O1 cholera and Nino3 were seen (Figure 8C). Some non-stationary (non-consistent over time) seasonal cycles were seen between O1 cholera and SSTs, rainfall, temperature and river levels in Matlab (Figure 8E, 8G, 7I and 7K). In general, the association of O139 cholera with all the environmental parameters was weaker than the association of O1 cholera with the same parameters.

Overall, the results of the analysis for cholera hospitalizations for all El Tor strains and for a classical biotype of *V. cholerae* were similar to the result for the El Tor O1 biotype strain alone because the frequency of the O1 biotype was dominant during the study period (see Supplementary online material, Figures S1–S5).

## Discussion

Our analyses indicated that the links between cholera hospitalizations and both IOD and the ENSO were not consistent overtime and that the timing of the associations was different between urban Dhaka and rural Matlab. In Matlab, the effect of ENSO on cholera hospitalizations was more dominant; there was no similar evidence of an effect of IOD on cholera hospitalizations.

The ENSO has been reported to play an important role in the interannual variation of endemic cholera in Bangladesh [6], [8]–[10]. There is evidence that interannual variability of cholera incidence between 1980 and 1998 in Dhaka is positively associated with average sea surface temperature (SST) in the Niño 3.4 region [9]. These studies assumed that the association of the ENSO with cholera incidence was consistent over the study period. However, our findings suggest that the previous findings based on this assumption can be improved by assuming a non-stationary (not consistent overtime) association, and by more accurately evaluating the possibly non-linear association between climatic covariates and cholera incidence.

It has been suggested that the IOD strongly influences sea level variations in the Bay of Bengal and sea levels have been linked with water levels in areas affected by tidal levels [23]. Because high sea levels in the eastern equatorial ocean are generally associated with a negative dipole in the tropical Indian Ocean, it follows that negative dipole events would tend to enhance flooding in Bangladesh and our results indicate that cholera incidence increases with enhanced flooding. The IOD also plays an important role as a modulator of the Indian monsoon rainfall. Ajayamohan and Rao reported a notable regime shift in the number of extreme rainfall events in the Ganges-Mahanadi Basin in India in the early 1980s, suggesting that the rainfall was modulated by coupled ocean-atmosphere conditions associated with a positive IOD [24]. Although causal pathways may be very complicated, flooding and drought are likely to be one of the important causal pathways for the observation that cholera hospitalizations peak both post-monsoon and pre-monsoon [28]. Flooding adversely affects water sources and sewerage systems increasing the exposure of populations to water contaminated with *V. cholerae*. The possible link between flooding and cholera may also be associated with the growth and multiplication of *V. cholerae*, because flooding can increase the level of insoluble iron which, in turn, improves the survival rate of *V. cholerae* [14]. It has also been suggested that flooding washes away the vibriophages that prey on *V. cholerae*, resulting in increased concentrations of the bacteria in the water [43]; a recent simulation study, however, does not support this hypothesis [44]. Low river discharge during the dry season may result in the intrusion of a salinity front and planktons towards the inland freshwater in the estuarine region, consequently providing the optimum environment for *V. cholerae* growth [28], [45].

It has been suggested that the effect of IOD on cholera incidence may be partially explained by the effect of IOD on SST and sea surface height (SSH) in the Bay of Bengal [30] and we found that, in 1998, high SSTs preceded a sharp increase in the number of cholera hospitalizations in Dhaka. In the Bay of Bengal, rising SSTs have been linked to higher concentrations of chlorophyll, a proxy for phytoplankton abundance [12]. High concentrations of phytoplankton may lead to high numbers of cholera-containing copepods, increasing the likelihood of cholera epidemics in coastal human populations [3]. However, a recent study reported that high river discharge in which large amounts of terrestrial nutrients are carried could be the main mechanism for a positive SST-phytoplankton relationship in the Bay of Bengal [46]. Investigations into the detailed pathways of the IOD-cholera relationship, particularly the role of SSTs and river discharges, are thus warranted.

The association between Dipole Mode Index (DMI) and cholera hospitalizations that we found in Dhaka was not observed in Matlab, while a consistent association between Nino3 and cholera hospitalizations was observed only in Matlab. The associations that we found between DMI and Nino3 with cholera are broadly consistent with previous finding [30]. A difference in the population density between Dhaka and Matlab may result in different transmission dynamics, hygiene and sanitation conditions, and behavioral patterns, some or all of which may explain the contradictory findings in these two areas [47]. Further work to clarify the role of oceanographic and local hydroclimatological phenomena in both these areas would be of interest.

There are some limitations in this study. First, less severe cases of cholera are less likely to have been included in the surveillance data; however, this should not pose a threat to the validity of the comparison over time, which was the subject of this study. Second, some cases could have been missed because of limitations in the capacity of the hospitals to receive the patients; particularly, during epidemics of cholera. However, in principle, the hospitals accept all patients who visit the hospital. Thus, the capacity of the hospitals should not be an important limitation of this study. Third, the wavelet analysis explores a periodical synchronicity between two time series, and so has no bearing on mechanisms. Further environmental and microbiological studies are necessary to elucidate the causal pathways of the associations.

Because of the serious global consequences of cholera and its sensitivity to climate, the World Health Organization has proposed that an early warning system for cholera epidemics using climatic parameters could be developed [48]. However, no highly accurate climate-based prediction system of cholera epidemics is currently available [49], [50]. A system for forecasting IOD has been developed, and IOD events are predictable up to 4 months in advance [51]. The results of our study call for the appropriate modeling of associations between cholera incidence and climatic factors, as has been done for other neglected tropical diseases, where non-stationary associations have been carefully handled [52] by incorporating non-linear functions to capture the non-stationary associations between climatic factors and population dynamics [53], [54]. All these efforts will provide the basis for accurately predicting cholera epidemics in Bangladesh, potentially improving the outcome for disease control efforts.

## Supporting Information

Figure S1 **Monthly time series of cholera hospitalizations by (a) serotype O1 & O139 and (b) bio-type El Tor & Classical for Dhaka (January 1983–December 2008).**
(TIF)

Figure S2 **Monthly time series of cholera hospitalizations by (a) serotype O1 & O139 and (b) bio-type El Tor & Classical for Matlab (November 1981–December 2008).**
(TIF)

Figure S3 **Cross wavelet coherence of global and local climatic time series with cholera in Dhaka and Matlab.** (A) Dipole mode index (DMI) and cholera in Dhaka; (B) DMI and cholera in Matlab; (C) Nino3 and cholera in Dhaka; (D) Nino3 and cholera in Matlab; (E) Sea surface temperature (SST) in the Bay of Bengal (°C) and cholera in Dhaka; (F) SST in the Bay of Bengal (°C) and cholera in Matlab; (G) Rainfall (mm) and cholera in Dhaka; (H) Rainfall (mm) and cholera in Matlab; (I) Temperature (°C) and cholera in Dhaka; (J) Temperature (°C) and cholera in Matlab; (K) River level (m) and cholera in Dhaka; (L) River level (m) and cholera in Matlab. Time series span from January 1983 to December 2008.
(TIF)

Figure S4 **Cross wavelet coherence of global and local climatic time series with classical biotype and El Tor cholera in Dhaka.** (A) Dipole mode index (DMI) and classical cholera; (B) DMI and El Tor cholera; (C) Nino3 and classical cholera; (D) Nino3 and El Tor cholera; (E) Sea surface temperature (SST) in the Bay of Bengal (°C) and classical cholera; (F) SST in the Bay of Bengal (°C) and El Tor cholera; (G) Rainfall (mm) and classical cholera; (H) Rainfall (mm) and El Tor cholera; (I) Temperature (°C) and classical cholera; (J) Temperature (°C) and El Tor cholera; (K) River level (m) and classical cholera; (L) River level (m) and El Tor cholera. Time series span from January 1983 to December 2008.
(TIF)

Figure S5 **Cross wavelet coherence of global and local climatic time series with classical biotype and El Tor cholera in Matlab.** (A) Dipole mode index (DMI) and classical cholera; (B) DMI and El Tor cholera; (C) Nino3 and classical cholera; (D) Nino3 and El Tor cholera; (E) Sea surface temperature (SST) in the Bay of Bengal (°C) and classical cholera; (F) SST in the Bay of Bengal (°C) and El Tor cholera; (G) Rainfall (mm) and classical cholera; (H) Rainfall (mm) and El Tor cholera; (I) Temperature (°C) and classical cholera; (J) Temperature (°C) and El Tor cholera; (K) River level (m) and classical cholera; (L) River level (m) and El Tor cholera. Time series span from November 1981 to December 2008, with the exception of river level which begins in January 1983.
(TIF)
