# Supplementary figures and images for: A Differential Effect of Indian Ocean Dipole and El Niño on Cholera Dynamics in Bangladesh
Source: PLoS One. 2013 Mar 29;8(3):e60001. doi: 10.1371/journal.pone.0060001 (PMC3612031; doi:10.1371/journal.pone.0060001)

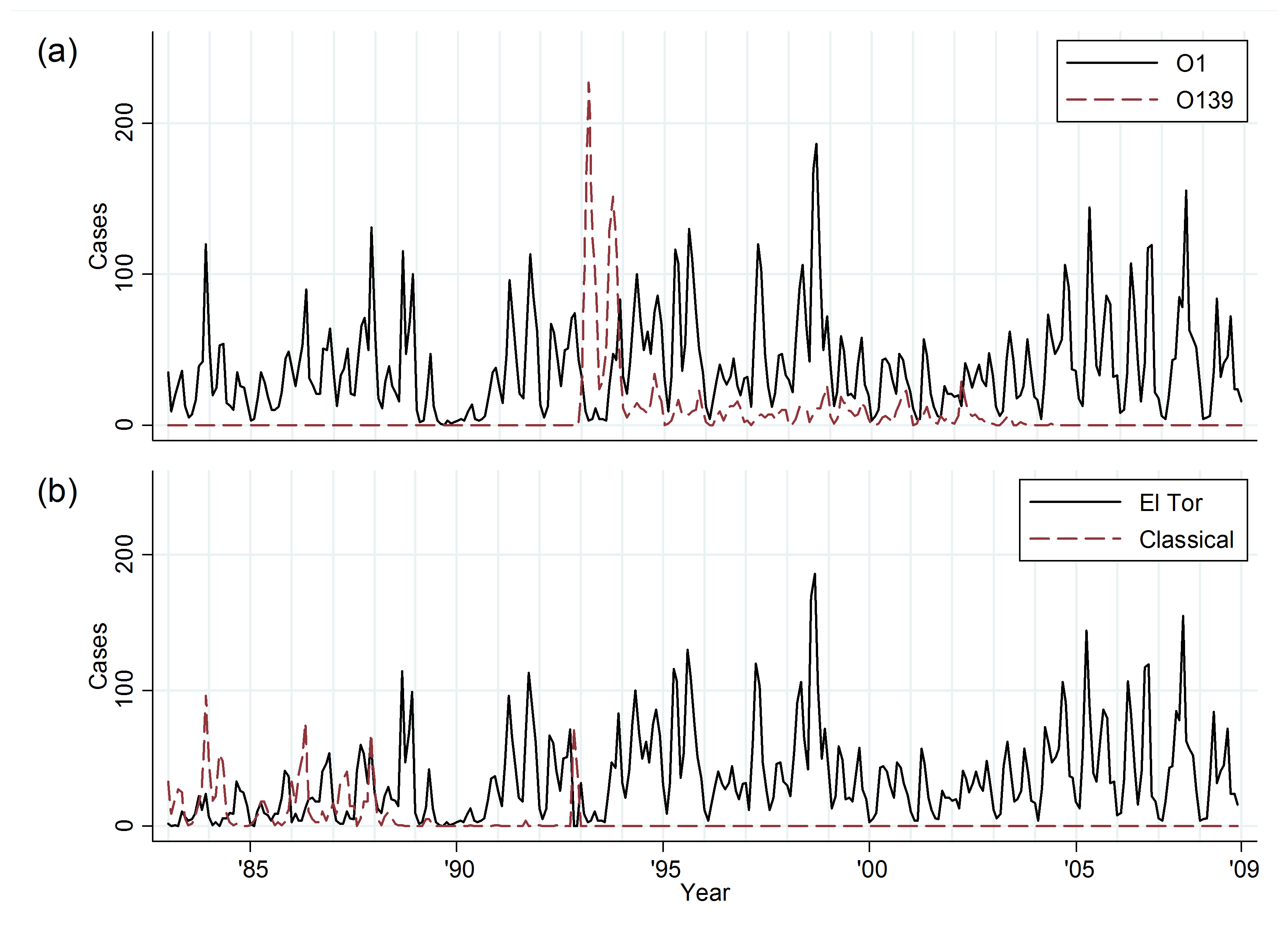

Supplement: Figure S1 — Monthly time series of cholera hospitalizations by (a) serotype O1 & O139 and (b) bio-type El Tor & Classical for Dhaka (January 1983–December 2008). (TIF) [file pone.0060001.s001.tif]

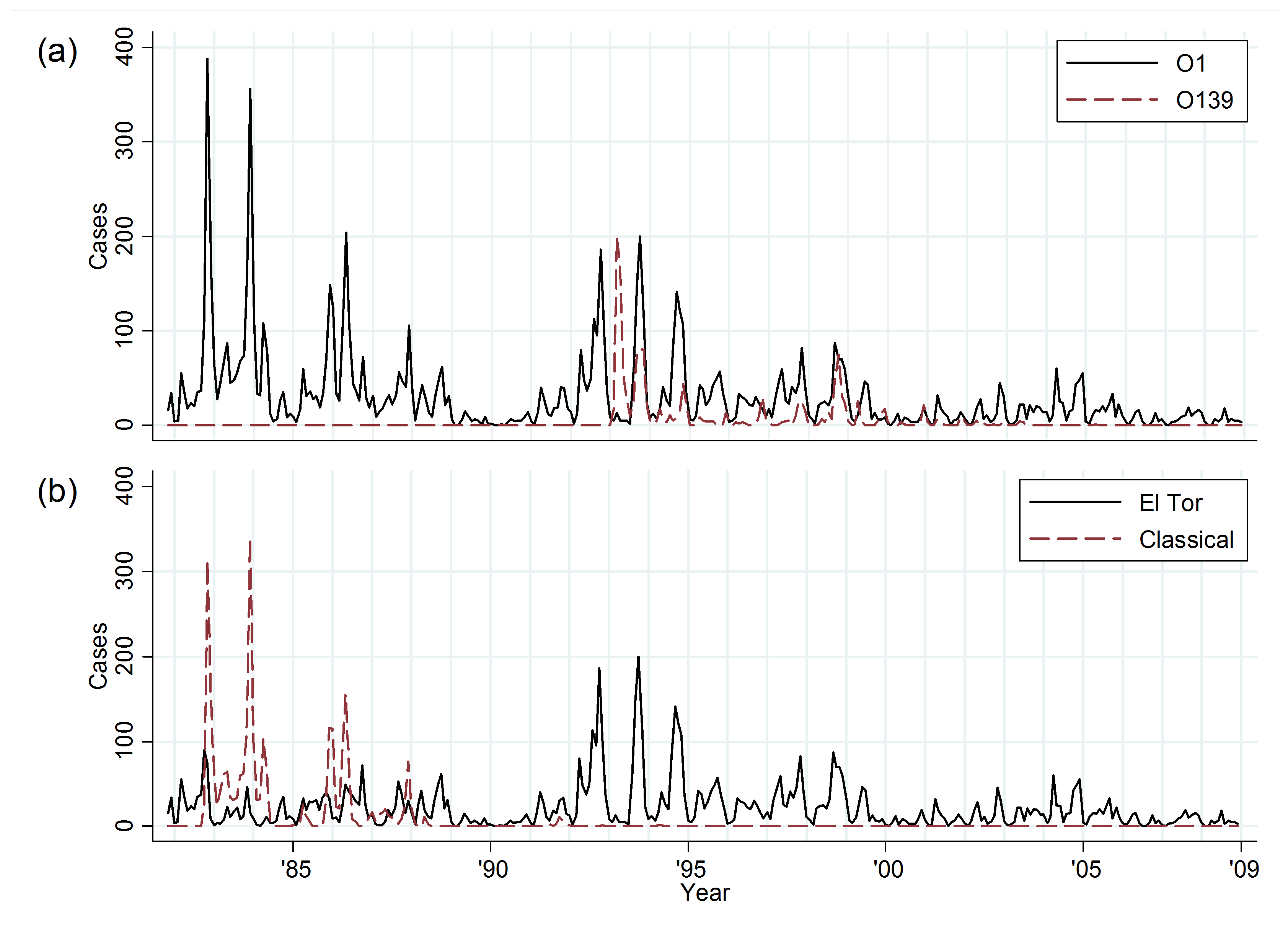

Supplement: Figure S2 — Monthly time series of cholera hospitalizations by (a) serotype O1 & O139 and (b) bio-type El Tor & Classical for Matlab (November 1981–December 2008). (TIF) [file pone.0060001.s002.tif]

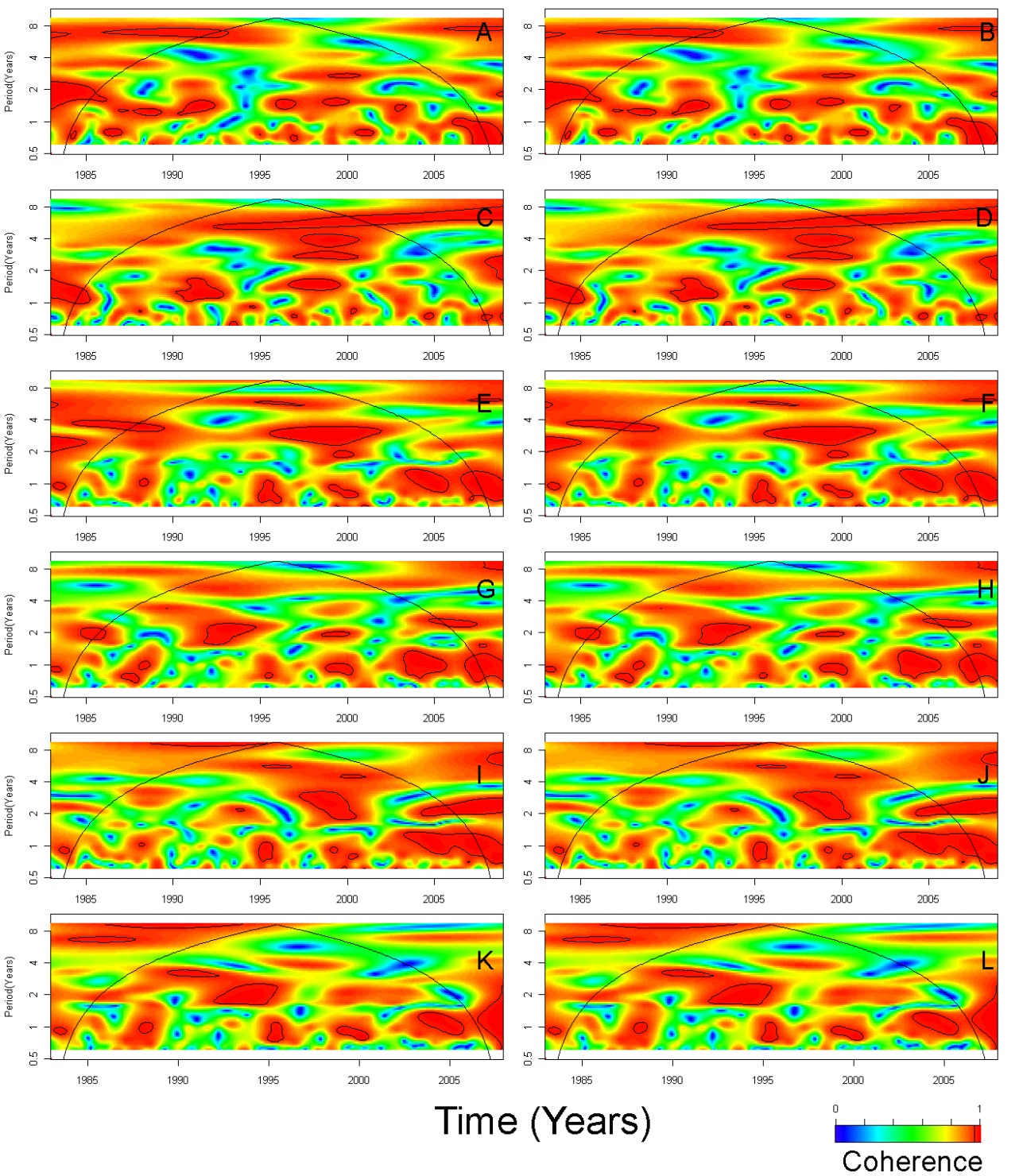

Supplement: Figure S3 — Cross wavelet coherence of global and local climatic time series with cholera in Dhaka and Matlab. (A) Dipole mode index (DMI) and cholera in Dhaka; (B) DMI and cholera in Matlab; (C) Nino3 and cholera in Dhaka; (D) Nino3 and cholera in Matlab; (E) Sea surface temperature (SST) in the Bay of Bengal (°C) and cholera in Dhaka; (F) SST in the Bay of Bengal (°C) and cholera in Matlab; (G) Rainfall (mm) and cholera in Dhaka; (H) Rainfall (mm) and cholera in Matlab; (I) Temperature (°C) and cholera in Dhaka; (J) Temperature (°C) and cholera in Matlab; (K) River level (m) and cholera in Dhaka; (L) River level (m) and cholera in Matlab. Time series span from January 1983 to December 2008. (TIF) [file pone.0060001.s003.tif]

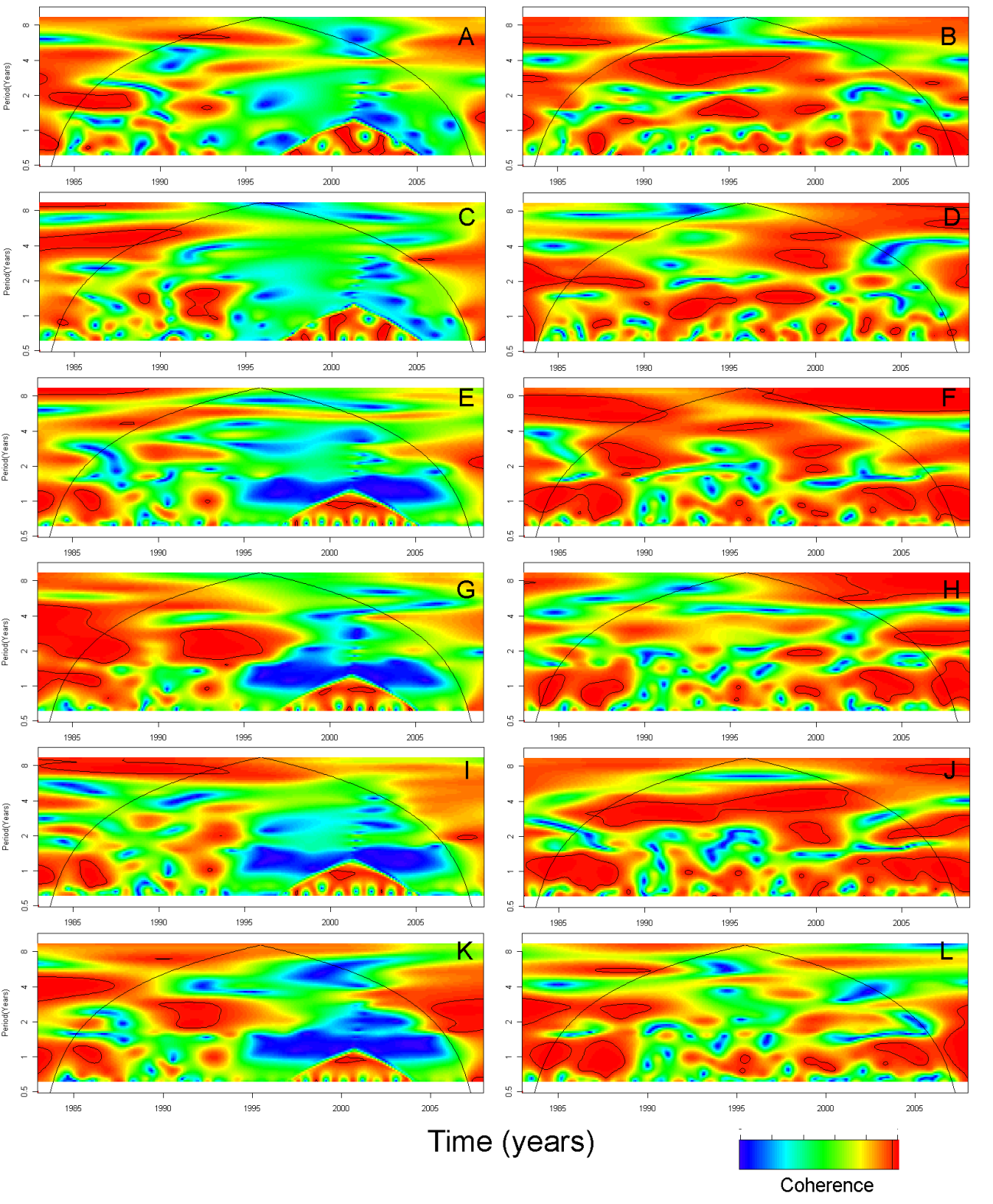

Supplement: Figure S4 — Cross wavelet coherence of global and local climatic time series with classical biotype and El Tor cholera in Dhaka. (A) Dipole mode index (DMI) and classical cholera; (B) DMI and El Tor cholera; (C) Nino3 and classical cholera; (D) Nino3 and El Tor cholera; (E) Sea surface temperature (SST) in the Bay of Bengal (°C) and classical cholera; (F) SST in the Bay of Bengal (°C) and El Tor cholera; (G) Rainfall (mm) and classical cholera; (H) Rainfall (mm) and El Tor cholera; (I) Temperature (°C) and classical cholera; (J) Temperature (°C) and El Tor cholera; (K) River level (m) and classical cholera; (L) River level (m) and El Tor cholera. Time series span from January 1983 to December 2008. (TIF) [file pone.0060001.s004.tif]

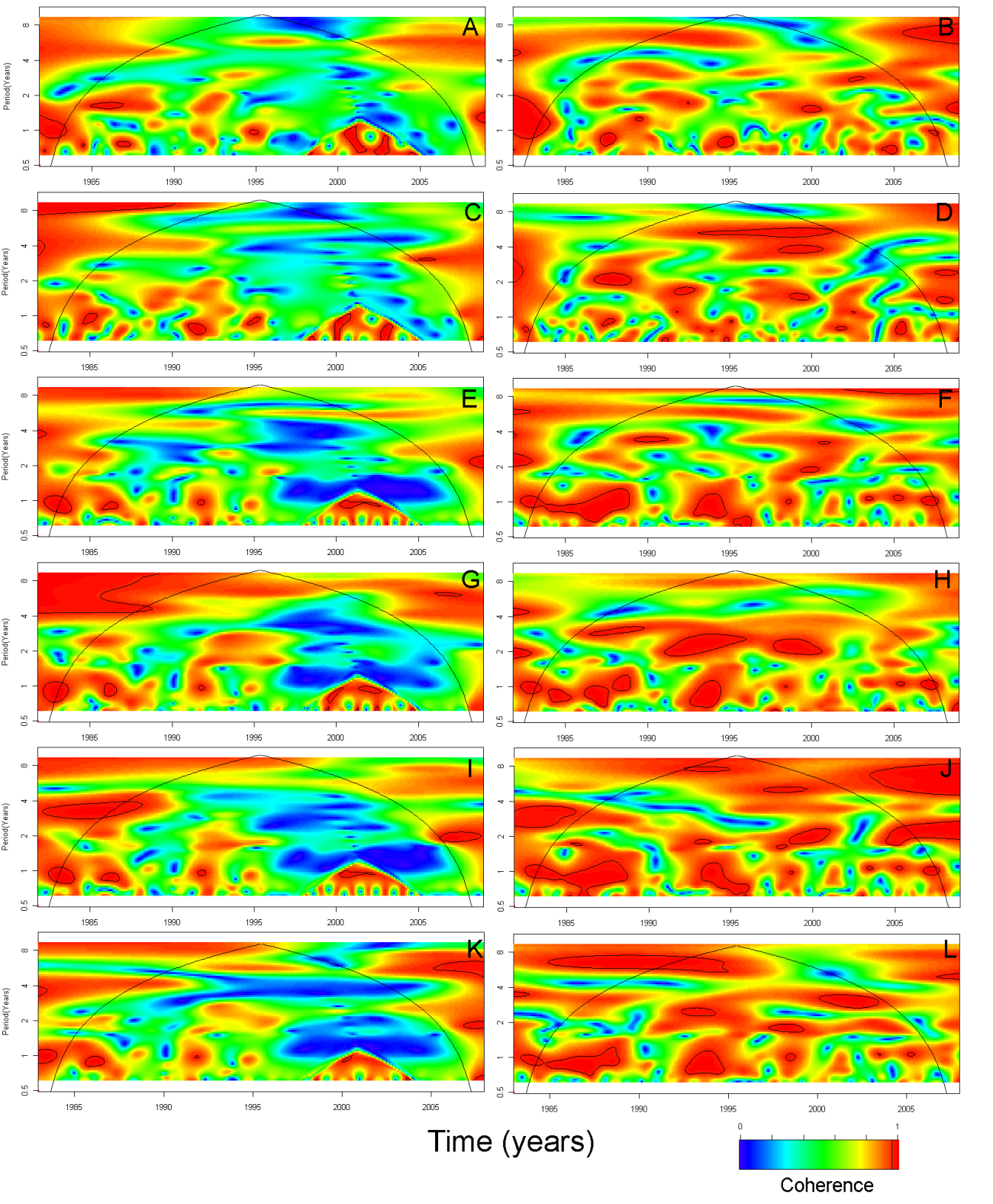

Supplement: Figure S5 — Cross wavelet coherence of global and local climatic time series with classical biotype and El Tor cholera in Matlab. (A) Dipole mode index (DMI) and classical cholera; (B) DMI and El Tor cholera; (C) Nino3 and classical cholera; (D) Nino3 and El Tor cholera; (E) Sea surface temperature (SST) in the Bay of Bengal (°C) and classical cholera; (F) SST in the Bay of Bengal (°C) and El Tor cholera; (G) Rainfall (mm) and classical cholera; (H) Rainfall (mm) and El Tor cholera; (I) Temperature (°C) and classical cholera; (J) Temperature (°C) and El Tor cholera; (K) River level (m) and classical cholera; (L) River level (m) and El Tor cholera. Time series span from November 1981 to December 2008, with the exception of river level which begins in January 1983. (TIF) [file pone.0060001.s005.tif]
